# Fuziline Ameliorates Glucose and Lipid Metabolism by Activating Beta Adrenergic Receptors to Stimulate Thermogenesis

**DOI:** 10.3390/ijms24098362

**Published:** 2023-05-06

**Authors:** He Gao, Zhenqiang Li, Chuanjing Cheng, Jing Cui, Jiamin Peng, Xiaoying Wang, Man Zhang, Yuanyuan Hou, Gang Bai

**Affiliations:** 1 State Key Laboratory of Medicinal Chemical Biology, College of Pharmacy and Tianjin Key Laboratory of Molecular Drug Research, Nankai University, Tianjin 300353, China; 2 School of Chinese Materia Medica, Tianjin University of Traditional Chinese Medicine, Tianjin 300193, China

**Keywords:** *Radix aconiti carmichaeli*, fuziline, thermogenesis, beta adrenergic receptors

## Abstract

*Radix aconiti carmichaeli* is a widely used traditional Chinese medicine that has been found to be effective in treating cardiovascular diseases and metabolic disorders. Patients with these diseases often experience a heat generation disorder, which is characterized by chilliness and can worsen the progression of the disease. This study established an in vitro screening model combining the examination of cellular mitochondrial membrane potential and mitochondrial temperature to screen drugs with thermogenic activity. After differentiation and determination of the content of characteristic metabolites of the drug-containing serum blood components, it was found that Fuziline (FZL) is the key thermogenic property in *Radix aconiti carmichaeli*, responsible for its thermogenic effects with a high relative importance of 33%. Experiments were conducted to evaluate the thermogenic activity of *Radix aconiti carmichaeli* and FZL in vivo by assessing temperature changes in various organs, including the rectum, liver, and brown adipose tissue. Moreover, the effects of intracellular β_3_-adrenergic receptor (β_3_-AR) agonistic effects were evaluated using transient β_3_-AR transfection and dual-luciferase assay systems. The molecular mechanism by which FZL promotes thermogenesis and improves mitochondrial function was investigated by verifying the β-adrenergic receptors (β-AR) downstream signaling pathway. The results suggest that FZL activates β-AR nonselectively, which in turn activates the downstream cAMP-PKA signaling pathway and leads to an increase in liver glycogenolysis and triglyceride hydrolysis, accompanied by enhancing mitochondrial energy metabolism. Consequently, the liver and brown adipose tissue receive energy to generate heat. In summary, these findings provide insight into the therapeutic application of *Radix aconiti carmichaeli* for metabolic disorders associated with heat generation disorders.

## 1. Introduction

*Radix aconiti carmichaeli* is a traditional herbal remedy that has been used for centuries to treat various human disorders, including metabolic diseases linked to a chilly sensation, such as hypothyroidism, adrenal suppression, heart failure, chronic renal failure, and so on [1]. Futhermore, *Radix aconiti carmichaeli* has been found to cure hypothermia [2] and treat mitochondrial abnormalities and energetic dysfunction in chronic renal failure [3,4,5,6]. Alkaloids are the major source of its toxicity and pharmacological activity. Studies have shown that methanolic extracts and alkaloid fractions of *Radix aconiti carmichaeli* can improve basal body temperature and mitochondrial dysfunction in heart failure animals, increase the content and activity of Na^+^-K^+^-ATPase and succinate dehydrogenase, and improve energy metabolism [7,8,9,10]. However, the mechanism by which *Radix aconiti carmichaeli* induces thermogenesis and cures mitochondrial abnormalities is still unclear.

Mammals exhibit three subtypes of adaptive thermogenesis, including shivering thermogenesis, non-shivering thermogenesis (NST), and diet-induced thermogenesis [11]. NST primarily occurs in skeletal muscle, liver, and brown adipose tissue (BAT) [12]. Among adult rats, muscle accounts for less than 50% of NST, while the liver, digestive tract, and BAT account for about 25%, 10%, and 10%, respectively [13,14]. In contrast, heart, brain, and white adipose tissue contribute less than 5% to NST. Numerous intracellular mechanisms participate in physiological thermogenic processes. In organisms, thermogenesis arises from the turnover of adenosine triphosphate (ATP) and adenosine diphosphate (ADP). Intracellular thermogenic processes, such as substrate cycling, ion cycling, and mitochondrial proton leaks, increase mitochondrial thermogenesis via increasing ATP utilization or decreasing ATP synthesis [15]. Sarcolipin-mediated uncoupling of Ca^2+^ cycling in the sarcoplasmic reticulum results in increased ATP consumption and subsequent thermogenesis [16,17]. As proton leakage continues, mitochondrial oxidative respiration occurs independently of ATP synthesis, and the lower the coupling efficiency, the higher the mitochondrial heat production [18].

The mobilization of energy reserves for thermogenesis in cells involves the release of liver glycogen and endogenous glycogen from cardiac and skeletal muscle as glucose, and the liberation of white adipose tissue triglycerides and endogenous triglycerides from brown adipose tissue as free fatty acids (FAs). Lipid-metabolism-mediated non-shivering thermogenesis (NST) is strongly linked to brown adipose tissue (BAT), which is rich in mitochondria [19]. FAs, the preferred energy substrate during thermogenesis, are generated from intracellular triglyceride lipolysis within the lipid droplets of BAT or taken up from circulating TG-rich lipoprotein (TRL)-derived FAs from the plasma by the enzyme lipoprotein lipase (LPL), which is highly abundant in BAT [20]. LPL gene expression and activity markedly increase in BAT upon activation of NST [21], causing a concomitant increase in TRL-derived fatty acid uptake, which accounts for approximately 50% of the whole-body TRL clearance [22]. The process of lipolysis and thermogenesis in white and brown adipocytes is mediated by β_3_-AR [23]. In response to β_3_-AR activation, cAMP-dependent protein kinase (PKA) phosphorylates hormone-sensitive lipase (HSL), which accelerates FA production and promotes the expression of thermogenic genes such as UCP1 [24]. FAs are oxidized to generate an electrochemical proton gradient in the mitochondrial inner membrane [25]. FAs activate uncoupling protein 1 (UCP1), which changes the protein conformation and promotes UCP-1-dependent protein leak, dissipating the mitochondrial proton motive force and promoting thermogenesis [23,26].

Glucose produced from glycogenolysis is another important source of energy for adaptive thermogenesis. To enhance glucose utilization, the glucose transporter proteins GLUT1 and GLUT4 facilitate glucose transport into cells for glycolysis. The activation of the PI3K-mTOR-Akt-Glut pathway promotes glucose uptake into BAT and skeletal muscle, where glucokinase rapidly phosphorylates glucose to generate pyruvate via the glycolytic pathway. Pyruvate is then oxidized to acetyl CoA in the mitochondria via the tricarboxylic acid cycle, which drives oxidative phosphorylation to produce ATP [27]. Additionally, citrate formed during this process can be utilized for de novo lipogenesis to replenish intracellular triglyceride stores in BAT during thermogenesis, thereby maintaining glucose-induced thermogenesis. This coordinated process ensures an adequate supply of energy substrates to sustain the thermogenic process and maintain body temperature in mammals.

In this study, we established an in vitro temperature screening model in BAT cells to evaluate the thermogenic potential of a drug-containing serum. By performing radial basis function (RBF) analysis, we identified Fuziline (FZL) as the thermogenic component of *Radix aconiti carmichaeli*. Subsequently, we confirmed the thermogenic activity of FZL both in vivo and in vitro. Furthermore, we investigated the thermogenesis mechanism of FZL and found that it modulatesβ-ARs to induce thermogenesis and regulate glucose and lipid metabolism. The present study firstly describes the mechanism and main pharmacological substances of FZ in the treatment of mitochondrial dysfunction and diseases with thermogenesis disorders, and proposes a new idea to improve mitochondrial function by agonizing β receptors (β-AR) to regulate febrile-disorder-related diseases. Our findings also provide a theoretical basis for the clinical treatment of metabolic disorders with *Radix aconiti carmichaeli*.

## 2. Results and Discussion

### 2.1. Radix Aconiti Carmichaeli Stimulated Thermogenesis via Activation of β-ARs

**Figure 1:**
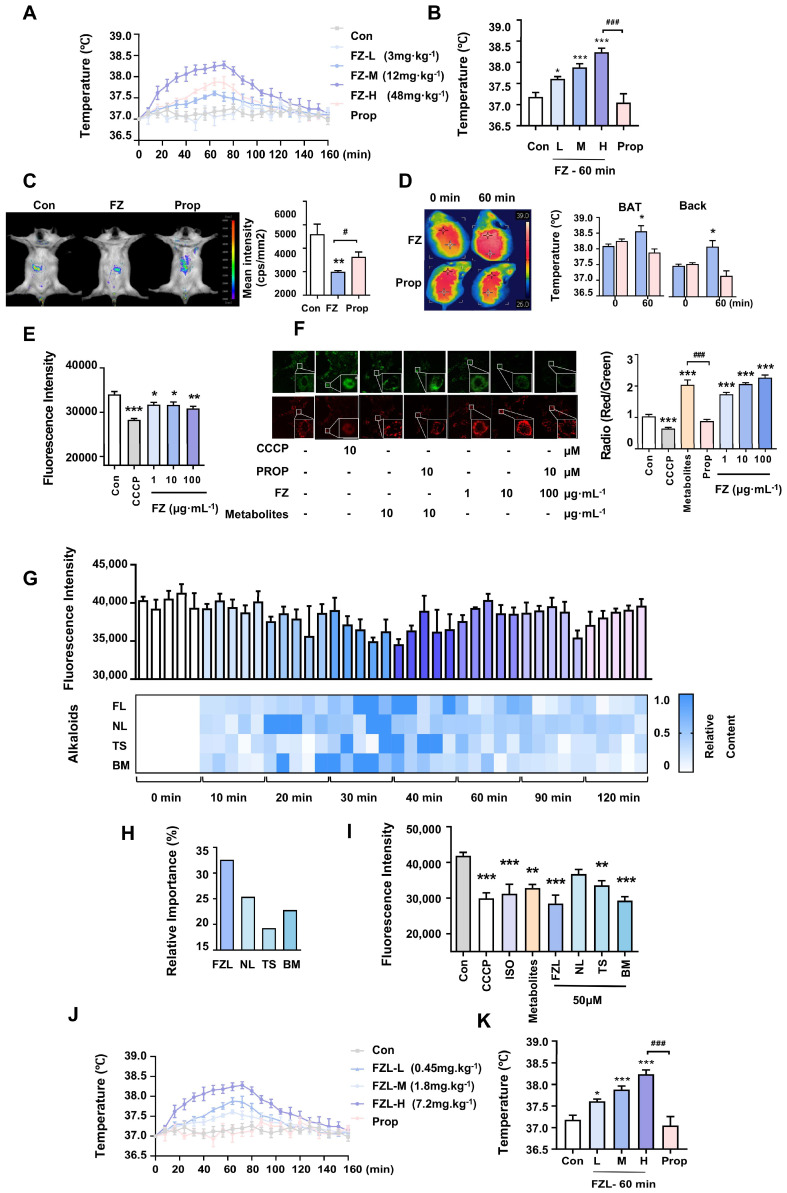
The thermogenic activity evaluation of FZ and thermogenic effectors screening. (**A**,**B**) Thermogenic activities of different concentrations of FZ in rats (60 min), (*n* = 5). (**C**) In vivo imaging assay of the RhBIV probe used for monitoring mouse liver temperature changes (*n* = 5). (**D**) Representative thermal images used for monitoring BAT temperature changes (*n* = 5). The effects of FZ on mitochondrial temperature (**E**) and MMP (**F**) (*n* = 3). (**G**) Evaluation of thermogenic activities of drug-containing serum and the relative content analysis of metabolite markers at different time points (*n* = 5). (**H**) The importance relationship between each metabolite marker and thermogenic activity obtained by RBF analysis, (*n* = 5). (**I**) The effects of metabolite markers on mitochondrial temperature (*n* = 3). (**J**,**K**) Thermogenic activities of different concentrations of FZL in rats (60 min), (*n* = 5). Significant differences between two groups were assessed using *t*-tests, and analysis of multiple groups was performed using one-way analysis of variance (ANOVA). * *p* <0.05, ** *p* < 0.01, *** *p* < 0.001 vs. Con group. ### *p* < 0.001 vs. metabolites group.

*Radix aconiti carmichaeli* exhibited positive effects on thermogenesis regulation in a clinical setting as a treatment for thermogenesis impairment in patients with hypothyroidism [28]. It is capable of treating chills. The chemical composition of the total extract of *Radix aconiti carmichaeli* was qualitatively analyzed by UPLC/Q-TOF-MS. A total of 23 components were identified by UPLC (Figures S1 and S2, Tables S1 and S2), shown in the supporting information. To investigate its potential activity on thermogenesis, the temperature-change effects of extracts of *Radix aconiti carmichaeli* (FZ) were detected in vivo. Specifically, we measured temperature changes in anal and liver tissues in rats and mice, respectively, following intragastric administration of FZ. Our findings, as shown in Figure 1, indicate that FZ administration resulted in a dose-dependent increase in anal temperature in rats (Figure 1A,B) and liver temperature in mice (Figure 1C). Notably, the temperature peaked at 60 min post administration and gradually returned to baseline levels thereafter. Furthermore, the administration of the β-AR inhibitor, Prop, effectively inhibited the FZ-induced increase in temperature. In addition to the liver, brown adipose tissue (BAT) is also a major organ involved in non-shivering thermogenesis (NST), particularly in rodents, where β3-ARs are essential for thermogenesis. Our results showed that the BAT temperature of mice treated with FZ was significantly higher than that of mice co-treated with Prop (Figure 1D). Based on these findings, we propose that FZ promotes adaptive thermogenesis through the activation of β-ARs in vivo.

The increase in liver temperature following FZ administration indicates increased mitochondrial heat production. To further validate the thermogenic effects of FZ in vitro, we measured changes in mitochondrial membrane potential (MMP) and mitochondrial temperature in brown fat cells (BFCs) in response to FZ treatment. Our results showed that FZ increased MMP and mitochondrial temperature in BFCs at various concentrations, while these effects were abolished by treatment with Prop (Figure 1E,F).

### 2.2. FZL Is the Key Bioactive Compound in FZ That Promotes Thermogenesis

In our previous results, ten metabolite markers of Aconite extract in blood were identified, of which four alkaloids, including FZL, neoline, talatisamine, and benzoylmesaconine, were screened for β-AR agonist [29]. We performed an analysis of serum-containing components at different time points after oral administration of FZ to analyze the variation in the contents of four alkaloids in the blood. To identify the thermogenic compounds in FZ, we established an in vitro mitochondrial temperature screening model and performed radial basis function (RBF) analysis. This analysis revealed a correlation between the four alkaloid metabolite markers, including FZL, neoline, talatisamine, and benzoylmesaconine, and thermogenic activity. The highest blood concentrations of all four metabolite markers were observed at the 30 min time point, which significantly stimulated thermogenesis in serum compared to other time points (Figure 1G). The performed radial basis function (RBF) analysis, to analyze the relationship between four alkaloids contents and thermogenic activity, demonstrated that FZL highly correlated with improved thermogenic activity (Figure 1H). The effects of the four metabolite markers on mitochondrial thermogenesis were assessed and the results indicated that FZL was the potential key bioactive compound for promoting thermogenesis (Figure 1I). Therefore, FZL was proven to be the potential key bioactive compound for promoting thermogenesis and further mechanism studies were carried out on it.

### 2.3. FZL Stimulated Thermogenesis through Activation of β_3_-ARs

**Figure 2:**
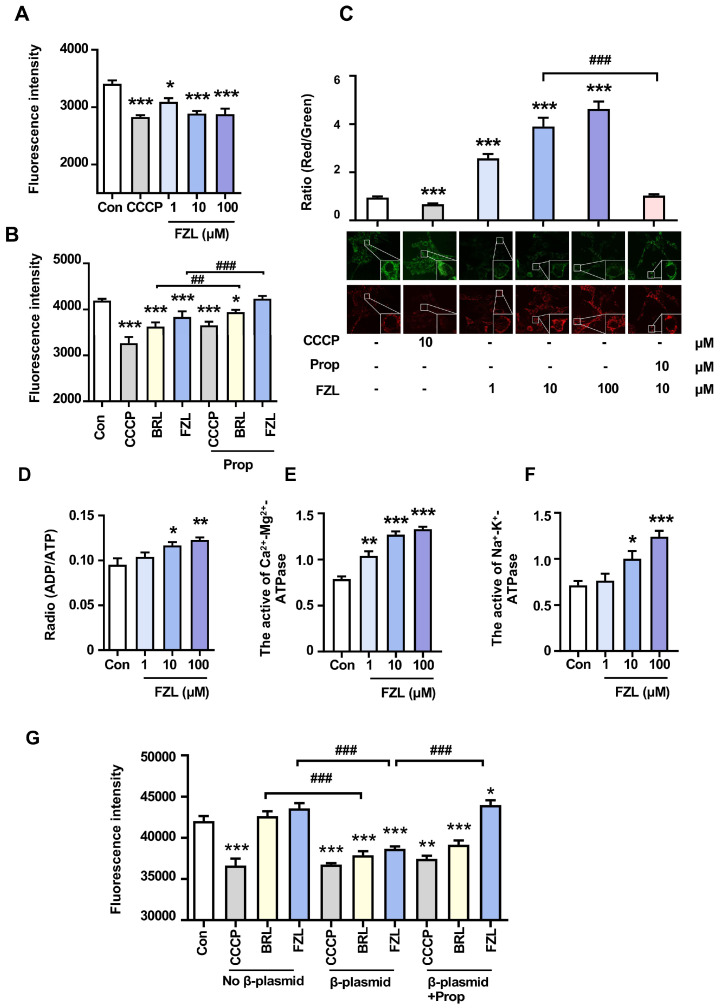
FZL stimulated thermogenesis through activation of β3-ARs. The relative effects of FZL on mitochondrial temperature (**A**,**B**) and MMP (**C**) were tested (*n* = 3). The effects of FZL on ADP/ATP radio (**D**) and enzyme activities of ATPase (**E**,**F**). (**G**) The relative changes in mitochondrial temperature of different transfected 293 cell lines upon FZL or BRL treatment. Significant differences between two groups were assessed using *t*-tests, and analysis of multiple groups was performed using one-way analysis of variance (ANOVA). * *p* <0.05, ** *p* < 0.01, *** *p* < 0.001 vs. Con group. ## *p* < 0.01, ### *p* < 0.001 vs. relative BRL or FZL groups.

β_3_-AR is the predominant receptor expressed in brown adipose tissue and is critical for adaptive thermogenesis [23]. As shown in Figure 2A,B, different concentrations of FZL and a commercial selective β_3_-AR agonist BRL both increased the mitochondrial temperature of brown fat cells (BFCs), whereas these responses were attenuated after treatment with Prop. Additionally, FZL significantly increased mitochondrial membrane potential (MMP) (Figure 2C) and the ADP/ATP ratio (Figure 2D), while also improving ATPase activity (Figure 2E,F). These findings indicate that FZL can enhance mitochondrial viability, promote ATP consumption during energy metabolism, and subsequently increase metabolic heat production. To determine whether β_3_-ARs are involved in the thermogenic effects of FZL, mitochondrial temperature was measured in 293T or β_3_-293T cells. As shown in Figure 2G, the temperature of 293T cells did not significantly change upon treatment with BRL or FZL. In contrast, the temperature of β_3_-293T cells increased significantly upon treatment with BRL or FZL, but this effect was reversed by co-treatment with Prop. These results revealed that FZL activates β_3_-AR receptors, enhancing mitochondrial viability and increasing metabolic heat production, leading to thermogenic effects in brown fat cells.

### 2.4. FZL Stimulated BAT Thermogenesis via β3-AR-Mediated Lipolysis

**Figure 3:**
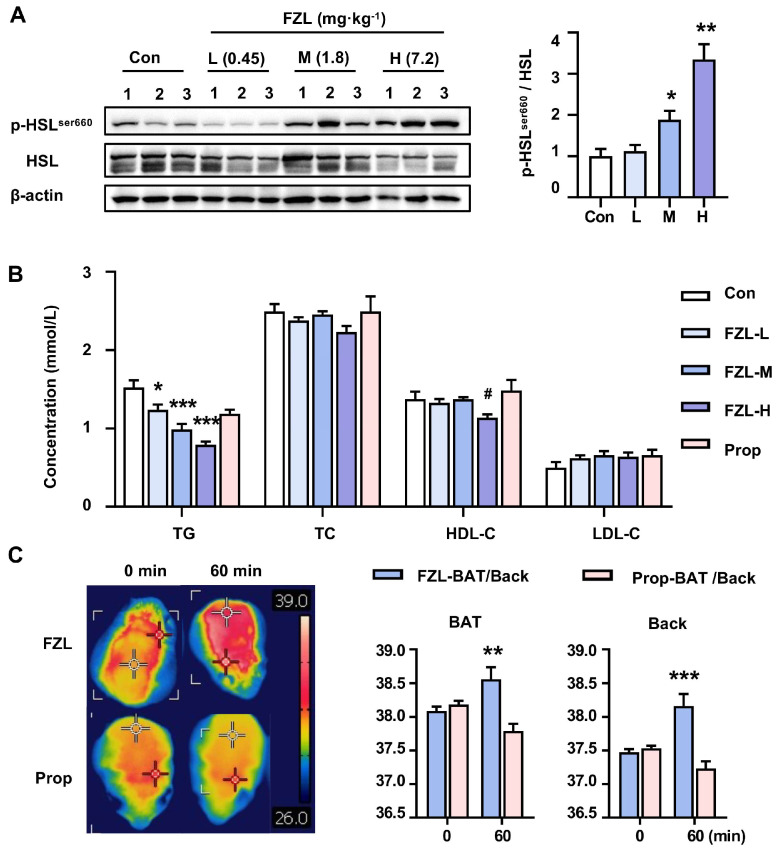
FZL stimulated BAT thermogenesis by promoting lipolysis. (**A**) FZL upregulated the phosphorylated levels of HSL (*n* = 3). (**B**) The contents of TG, TC, LDL-C, HDL-C in FZL-treated mice serum were analyzed by a Point care M3 biochemistry analyzer (*n* = 5). (**C**) Representative thermal images were used to monitor BAT temperature changes in mice (*n* = 5). Significant differences between two groups were assessed using *t*-tests, and analysis of multiple groups was performed using one-way analysis of variance (ANOVA). * *p* <0.05, ** *p* < 0.01, *** *p* < 0.001 vs. Con or FZL group. # *p* < 0.05 vs. Con group.

Upon activation of the β_3_-AR, the downstream cyclic adenosine monophosphate (cAMP) pathway is activated, leading to the activation of protein kinase A (PKA). PKA, in turn, phosphorylates hormone-sensitive lipase (HSL), the primary lipase in brown adipose tissue (BAT), which promotes lipolysis in BAT lipid droplets. Previous research demonstrated that *Radix aconiti carmichaeli* acts as a non-selective β-ARs agonist [30]. The extracts of *Radix aconiti carmichaeli* improved lung function in the early stage of pulmonary hypertension through the synergistic action of β-ARs signals [31]. Alcohol amine alkaloids in *Radix aconiti carmichaeli* acted as β-ARs agonists by increasing cAMP levels [32]. Increased responsiveness to cAMP levels was observed in a mouse model of thyroid function suppression and adrenal cortex suppression, and regulated the maximum binding capacity of β-ARs to treat renal failure [33]. Lower concentrations of the plant alkaloid mesaconitine can stimulate β-ARs [30]. To investigate the specific mechanisms through which Fuziline (FZL) regulates the thermogenic activity of BAT, the levels of phosphorylated HSL in BAT and plasma triglycerides (TGs) were measured. As demonstrated by Figure 3A,B, the levels of phosphorylated HSL were elevated and plasma TG levels were decreased in the FZL groups compared to the control group, and both of these FZL-induced effects were abolished by propranolol (Prop) treatment. FZL increased the production of free fatty acids (FFAs) through cellular lipolysis or uptake from the bloodstream. Increased FFAs in BAT as substrates for β-oxidation increased membrane potential in BAT and activated proton transport by the uncoupling proteins UCP1 to increase mitochondrial heat production in BAT [34]. Accordingly, FZL enhanced cellular energy metabolism in BAT, consistent with the findings in experimental cells, including improved ATPase activity, mitochondrial membrane potential (MMP), and mitochondrial temperature. As shown in Figure 3C, intragastric administration of FZL significantly raised BAT temperature, indicating that FZL stimulates BAT thermogenic activity in mice. This FZL-induced increase in BAT temperature was significantly attenuated by Prop. Taken together, FZL was found to have β_3_-ARs agonism in vivo and was suggested to promote thermogenesis in BAT through the regulation of β_3_-AR-mediated lipid metabolism.

### 2.5. FZL Accelerated Liver Glycogenolysis through Activation of β_2_-Adrenergic Receptor (β_2_-AR)

**Figure 4:**
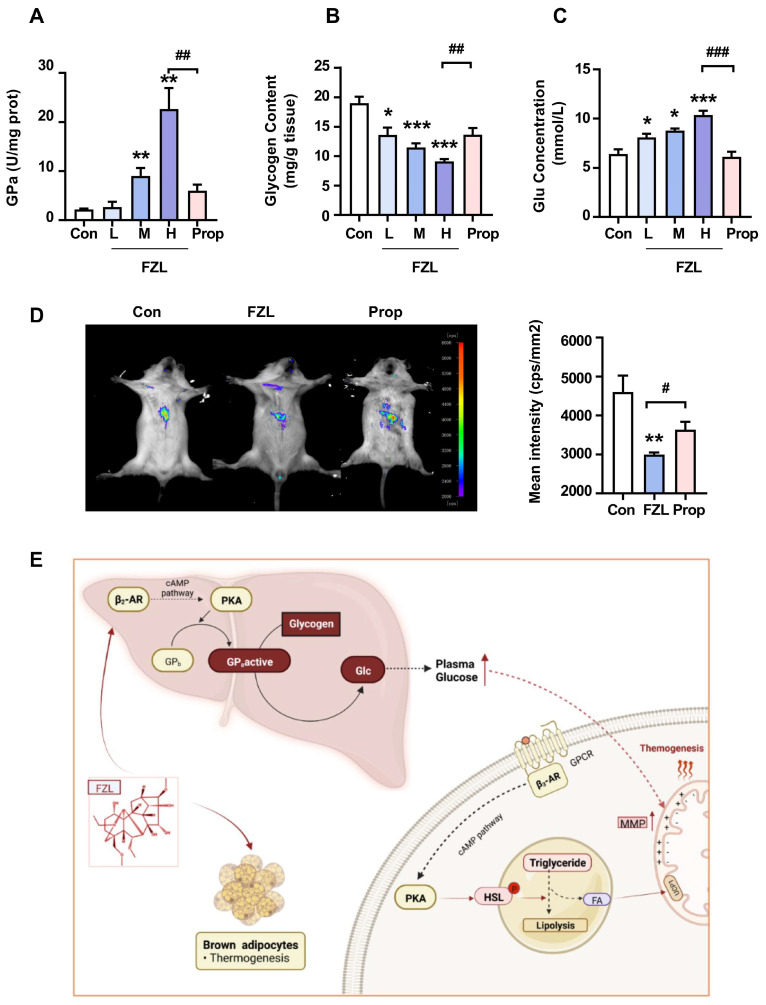
FZL accelerated thermogenesis by promoting liver glycogenolysis. The effects of FZL on GP enzyme activities (**A**), hepatic glycogen (**B**), and glucose levels (**C**) (*n* = 5). (**D**) In vivo imaging assay of the RhBIV probe used to monitor temperature changes in the liver of mice (*n* = 5). (**E**) Detailed diagram of the mechanism whereby FZL modulates β-ARs to regulate glucose and lipid metabolism for promoting thermogenesis. Significant differences between two groups were assessed using *t*-tests, and analysis of multiple groups was performed using one-way analysis of variance (ANOVA). * *p* < 0.05, ** *p* < 0.01, *** *p* < 0.001 vs. Con group. # *p* < 0.05, ## *p* < 0.01, ### *p* < 0.001 vs. relative FZL-H or FZL groups.

Various processed products of *Radix aconiti carmichaeli* improved cardiac function and vascular smooth muscle in rats with heart failure, which may be related to the upregulation of β_2_-AR mRNA expression in myocardial tissue [35]. Forty-five compounds working as β_2_-AR agonists were identified in *Radix aconiti carmichaeli*, mainly alkanolamine-diterpene alkaloids and monoester-diterpene alkaloids [36,37]. FZL has also been proven to a β_2_-AR agonist before [29]. The activation of β_2_-AR has been reported to accelerate liver glycogenolysis and increase plasma glucose levels, playing a crucial role in regulating blood glucose balance [38]. Glucose can fuel thermogenesis, with the exception of FFAs. Therefore, FZL was speculated to induce thermogenesis by activating β_2_-AR. To verify this presumption, we measured FZL-stimulated liver glycogenolysis. β_2_-ARs regulate the phosphorylation state of key regulatory enzymes and glycogen turnover via the adenylyl cyclase/cAMP-signaling pathway [12]. Hepatic GP is phosphorylated to GPa, a catalytically active form of GP, which catalyzes liver glycogenolysis. As shown in Figure 4A, FZL treatment significantly increased the content of hepatic GPa. Accordingly, hepatic glycogen levels decreased (Figure 4B) and plasma glucose levels increased (Figure 4C). FZL treatment also improved liver temperature in the mice (Figure 4D), which was consistent with the above observations, suggesting that FZL accelerated adaptive thermogenesis. β_2_-AR agonists can improve muscle glucose transport, and the agonism of β_2_-ARs on the liver stimulates hepatic glycogenolysis and increases plasma glucose. Glucose–insulin infusion increases oxygen consumption and energy expenditure in healthy subjects. This thermogenic response was largely blocked by propranolol, suggesting that it was partially mediated by β-Ars [38]. Above all, FZL agonizes both β_2_-ARs and β_3_-ARs and regulates glucose and lipid metabolism through downstream cAMP-signaling pathways, promoting adaptive thermogenesis as shown in Figure 4E. The activation of non-shivering thermogenesis in rats increases hepatic gluconeogenesis, hepatocyte mitochondrial respiration, and liver temperature [39]. The agonism of hepatic β_2_-ARs stimulates hepatic glycogenolysis and increases plasma glucose as an energy supply for thermogenesis. Moreover, the agonism of β_3_-AR in brown adipose tissue increases glucose utilization in adipose tissue and promotes lipid metabolism for thermogenesis [40,41]. Long-term use of CL-316243 or BRL37344 (selective β_3_-AR agonists) increased glucose uptake in white and brown adipocytes without stimulating GLUT4 translocation [42]. Thus, the interaction of β_2_-ARs and β_3_-ARs accelerates glucose utilization and thermogenesis. In summary, we suggested that FZL increases thermogenesis by agonizing β_2_-AR-stimulated glycogenolysis and β_3_-AR-stimulated lipid metabolism.

According to our study, FZL could treat impaired thermogenesis by activating BAT thermogenesis and hepatic glycogenolysis for energy supply. Short-term use of FZL has the effect of activating BAT thermogenesis without upregulation of UCP1 expression, which can avoid BAT atrophy caused by an overexpression of UCP1. In this process, FAs and blood glucose are applied as multiple energy supplies at the same time and result in high thermogenic efficiency. DIO2 is another key component of thermogenesis that is in fact required for the activation of thermogenesis and is activated by β-AR agonists [43,44,45]. Whether FZL affects the expression of UCP1 and DIO2 remains to be confirmed in the future. It is worth noting that *Radix aconiti carmichaeli* also exhibited potential toxicity, including cardiotoxicity, neurotoxicity, embryotoxicity, and nephrotoxicity [28]. Cases of poisoning by *Radix aconiti carmichaeli* present with a combination of neurological, cardiovascular, and gastrointestinal features [46]. Toxicological studies demonstrated that the acute cardiotoxicity and neurotoxicity of *Radix aconiti carmichaeli* are derived from the DDAs [10]. In order to reduce toxicity, the combination of *Radix aconiti carmichaeli* with other herbs in a formula can reduce its acute toxicity and maintain or even enhance its pharmacological activity. Ensuring the safe and effective use of *Radix aconiti carmichaeli* requires standardized detoxification methods and strict quality control.

## 3. Materials and Methods

### 3.1. Chemicals and Reagents

Black-processed pieces of *Radix aconiti carmichaeli* were obtained from Tongrentang Chinese Medicine Co., Ltd. (Beijing, China). FZL, purity > 98%, and neoline (NL, purity > 98%) were purchased from Yuanye Biotechnology Co., Ltd. (Shanghai, China). Talatisamine (TS, purity > 98%) and benzoylmesaconine (BM, purity > 98%) were purchased from Macklin Biotechnology Co., Ltd. (Shanghai, China). Carbonyl cyanide 3-chlorophenylhydrazone (CCCP), BRL37344 and Propranolol Hydrochloride (Prop) were purchased from Sigma-Aldrich Chemical Co., Ltd. (Shanghai, China). The CRE-Luc reporter plasmid pGL4.29 and the Renilla luciferase reporter vector plasmid pRL-TK were purchased from Promega Biotechnology Co., Ltd. (Madison, WI, USA). The β_3_-AR plasmid was purchased from OriGene Biotechnology Co., Ltd. (Wuxi, China). Primary antibodies against HSL (4107T), phospho-HSL^S660^ (45804S), β-actin (45804S), and secondary antibodies were purchased from Cell Signaling Technology Co., Ltd. (Beverly, MA, USA). All the reagents used in cell culture were purchased from Gibco BRL Life Technology Co., Ltd. (Grand Island, NY, USA).

### 3.2. Extracts of Radix Aconiti Carmichaeli Preparation

An amount of 3 g of black-processed pieces of *Radix aconiti carmichaeli* was crushed and placed in a conical flask, and 30 mL ethanol was added, which contained 0.05% HCl, for ultrasonic extraction at 30 °C for 40 min. After three repetitions, the extracts were combined and evaporated to dryness under reduced pressure with a rotary evaporator. Concentrated products were prepared, and named FZ for administration according to previous research [29].

### 3.3. UPLC-Q/TOF-MS Analysis of FZ

The chemical composition of the total extract of *Radix aconiti carmichaeli* was qualitatively analyzed by UPLC/Q-TOF-MS using mass spectrometer with an electrospray ionization (ESI) ionization source in positive mode. A C18 column (BEH, 2.1 × 100 mm, 1.7 μm; Waters, USA) was applied to separate the samples. The mobile phase was a gradient of A (0.1% formic acid/H2O) and B (CH3CN). Further details and analysis are detailed in the supporting information.

### 3.4. Animals

Male KM mice (18–22 g) and male SD rats (200–220 g) were purchased from Beijing Vital River Laboratory Animal Technology Co., Ltd. (Beijing, China). The animals were randomly assigned to cages with free access to water and standard mouse chow under 25 °C and adaptively fed for one week before experiment. All animal experiments were carried out in accordance with the National Institute of Health Guide for the Care and Use of Laboratory Animals and approved by the Nankai University of Laboratory Animals Care and Use Committee (TCM-LAEC2019013; Tianjin, China).

### 3.5. Anal Temperature Measurement in Rats

The rats were divided into 5 groups (*n* = 5): the Con group (i.g. saline), the FZ groups (i.g. 3, 12, or 48 mg·kg^−1^ FZ), and the Prop group (i.g. 48 mg·kg^−1^ FZ + i.p. 10 mg·kg^−1^ Prop). Before administration, the rats’ basal anal temperature was measured at room temperature. The anal temperature of rats was measured every 8 min for 2 h after drug administration via a BL-420 biological function experiment system (Taimeng, Chengdu, China), according to previous research [47].

### 3.6. Liver Temperature Measurement

The mice were shaved and divided into different groups (*n* = 5): the Con group (i.g. saline), the FZ group (i.g. 48 mg·kg^−1^ FZ), and the Prop group (i.g. 48 mg·kg^−1^ FZ + i.p. 10 mg·kg^−1^ Prop). Half an hour before administration, the mice were injected with a RhBIV probe (iv, 10 mg·kg^−1^) [48]. After 1 h of administration, the corresponding fluorescence intensity in vivo was detected through the small animal in vivo imager (NightOWL IILB 983, Berthold Technologies, Batterveldbad City, Germany).

### 3.7. Thermographic Imaging

The mice were divided into different groups (*n* = 5) and the hair of the interscapular and dorsal region was shaved. After administration, the thermal images were taken by an infrared radiation thermal imaging camera (FLIR, E60, Wilsonville, OR, USA) every 10 min for 2 h. Images were analyzed by FLIR Tools software (FLIR, E60, Wilsonville, OR, USA). The highest temperature values of the interscapular and dorsal region were used for analysis.

### 3.8. Cell Culture

The brown fat cells (BFCs) were purchased from the Procell Life Science&Technology Co., Ltd. (Wuhan, China) and were cultured in complete medium for primary cells (Procell Co., Wuhan, China). The human embryonic kidney cell line (293T) was purchased from American Type Culture Collection (Manassas, VA, USA) and was cultured in DMEM supplemented with 10% fetal bovine serum, 100 U/mL penicillin G, and 100 mg/mL streptomycin. β_3_AR transfected 293T (β_3_-293T) cells were constructed according to previous research [34]. All cells were incubated at 37 °C with 5% CO_2_ in a humidified incubator.

### 3.9. Mitochondrial Membrane Electrochemical Potential (MMP) and Temperature Assay

BFCs, 293T, and β_3_-293T cells were cultured in 6-well plates or 96-well plates and were pretreated with different compounds for 1 h or 50 mM CCCP for 30 min.

The BFCs cells, which were cultured in 6-well plates, were then stained with JC-1. MMP was quantified using the JC-1 detection kit, which was obtained from Leagene Biotechnology Co., Ltd. (Beijing, China). The fluorescence images were captured with a spectral-type LSM 700 confocal laser scanning microscope (Carl Zeiss, Oberkochen, Germany) and quantified according to previous research [49].

In addition, temperature changes in the mitochondria of cells were detected using a thermosensitive mitochondrial-targeted fluorescent probe Mito Thermo Yellow (MTY). The BFCs, 293T, and β_3_-293T cells, which were cultured in 96-well plates, were incubated with a 50 mM MTY probe for 20 min. After washing with PBS, the temperature change was detected using a constant temperature multimode reader (Spark 10 M, TECAN, Shanghai, China) according to previous research [49].

### 3.10. Metabolites Identification

The SD rats were fasted one night before the experiment and given intragastric administration of FZ (15 mg·kg^−1^). Subsequently, blood was taken at 0, 10, 20, 30, 40, 60, 90, and 120 min after administration. After deproteinization, the supernatant was divided into two parts in equal volume and exsiccated under nitrogen flow to obtain the metabolites. One part was redissolved with complete medium for the mitochondrial temperature experiment. The another was redissolved with methanol and 12,000 rpm centrifuged at 4 °C for 10 min. The supernatant was collected for UPLC-Q/TOF-MS analysis according to previous research [29]. An Acquity BEH C18 column (2.1 × 100 mm, 1.7 μm; Waters Co., Milford, MA, USA) was used for the separations. The injection volume was 1.0 μL, with a gradient elution of acetonitrile (A) and a 0.1% formic acid aqueous solution (B) using the following protocol: 2–15% A at 0–2 min, 15–18% A at 2–6 min, 18–22% A at 6–7 min, 22–35% A at 7–16 min, 35–55% A at 16–18 min, 55–100% A at 18–23 min, 100 % to 2% A at 23–25 min, and 2% A at 25–26 min. Full-scan data were gathered from 100 to 1500 Da in positive mode. All data were analyzed by Marker lynx software (v4.1, Water, Milford, MA, USA).

### 3.11. SPSS Analysis

The contents of four metabolite markers, including FZL, neoline, talatisamine, and benzoylmesaconine, were relatively quantified by extracting the normalized molecular ion peak intensities under cationic detection conditions. First, the ion flow of four metabolite markers was detected in blood samples to obtain the corresponding blood content at 0, 10, 20, 30, 40, 60, 90, and 120 min after administration. Second, the mitochondrial temperature change in BFCs was detected using MTY to investigate the bioactivity of drug-containing serum at different time points. The normalized total bioactivity values and the contents of the compounds at corresponding points were imported into IBM SPSS statistics software (version 16.0, Chicago, IL, USA) for correlation calculations. The Radial Basis Function (RBF) method, a feed-forward network with a single hidden layer, was applied to indicate the relationship between the content of each marker and thermogenic bioactivity.

### 3.12. ADP/ATP Ratio and ATPase Enzymatic Activity Assay in BFCs

BFCs were cultured in 20 mm culture dishes and incubated with different compounds for 1 h. BFCs were lysed with RIPA lysis buffer, and the cell lysates were added with corresponding reagents and heated immediately. The ADP/ATP ratio was measured by the Colorimetric-Fluorometric Assay Kit (Cell Signaling Co., CA, USA). The enzymatic activity of Na^+^-K^+^-ATPase and Ca^2+^-Mg^2+^-ATPase were determined by reagent kits (Solarbio Co., Beijing, China) according to previous research [50].

### 3.13. Detection of Intracellular and Circulating Lipolytic Activity

The mice were divided into 5 groups (*n* = 5): the Con group (i.g., saline), the FZL groups (i.g., 0.45, 1.8 or 7.2 mg·kg^−1^ FZL), and the Prop group (i.g. 7.2 mg·kg^−1^ FZL + i.p. 10 mg·kg^−1^ Prop). After 1 h of administration, blood samples were taken from the angular vein and plasma, and BAT and livers were collected for further biochemical indicators assay.

The expression levels of HSL, phosphorylated HSL (p-HSL^ser660^), and β-actin in BAT were analyzed by Western blot. The contents of triglycerides (TG), total cholesterol (TC), high-density lipoprotein cholesterol (HDL-C), and low-density lipoprotein cholesterol (LDL-C) in plasma were determined using the comprehensive test disks for plasma index assay (Dymind Co., Tianjin, China) according to previous research [51].

### 3.14. Detection of Liver Glycogenolysis Activity

The content of glycogen and the activity of glycogen phosphorylase (GP) in the liver of mice were determined by glycogen detection kit (Jiancheng Co., Nanjing, China) and GPa detection kit (Comin Co., Suzhou, China). The contents of glucose (Glu) in plasma were determined using the comprehensive test disks for plasma index assay (Dymind Co., Tianjin, China). The analysis protocols were followed according to previous research [52].

### 3.15. Statistical Analysis

All data are expressed as the mean values ± standard deviations (SD). Significant differences between two groups were analyzed by *t*-tests, and differences between multiple groups were analyzed by analysis of one-way ANOVA test using GraphPad Prism (version 9.0, San Diego, CA, USA). The post hoc test is Bonferroni’s multiple comparisons test. *p* < 0.05 was considered statistically significant.

## 4. Conclusions

In conclusion, our findings demonstrated that *Radix aconiti carmichaeli* and its representive thermally active component FZL promote adaptive thermogenesis through the activation of β-ARs in vivo and support the thermogenic effects of *Radix aconiti carmichaeli* in vitro using brown fat cells. These results provided insight into the potential use of *Radix aconiti carmichaeli* as a therapeutic agent for metabolic disorders characterized by impaired thermogenesis, which may help develop novel therapeutic strategies for metabolic disorders associated with heat generation disorders. This study laid a foundation for promoting the clinical application of *Radix aconiti carmichaeli*.

## Data Availability

The original contributions presented in the study are included in the article. Further inquiries can be directed to the corresponding author.

## Supplementary Materials

The following supporting information can be downloaded at: https://www.mdpi.com/article/10.3390/ijms24098362/s1.
