# Supplementary material for: Fuziline Ameliorates Glucose and Lipid Metabolism by Activating Beta Adrenergic Receptors to Stimulate Thermogenesis
Source: Int J Mol Sci. 2023 May 6;24(9):8362. doi: 10.3390/ijms24098362 (PMC10179377; doi:10.3390/ijms24098362)
Supplement: Supplementary file 1 [file ijms-24-08362-s001.zip › ijms-2330821-supplementary.pdf]

Supporting Information

Fuziline Ameliorates Glucose and Lipid Metabolism by Activating Beta Adrenergic Receptors to Stimulate Thermogenesis

1 UPLC-Q/TOF-MS analysis conditions

An UPLC system equipped with a Q/TOF-MS and electrospray ionization (ESI) source was used to analyze the samples. A C18 column (BEH, 2.1 × 100 mm, 1.7 µm; Waters, USA) was applied to separate the samples. The mobile phase was a gradient of A (0.1% formic acid/H2O) and B (CH3CN), conditions are described (**Table S1)** The flow rate was 0.40 mL/min, and the column temperature was maintained at 30 °C. PDA detection 190-400 nm scan was run and injection volume was 2.0 µL.

Table S1 Mobile phase elution conditions

Time(min)	Flow($mL\cdot min^{-1}$)	A(%)	B(%)	Curve
Initial	0.400	98.0	2.0	Initial
2.00	0.400	85.0	15.0	4
6.00	0.400	82.0	18.0	4
7.00	0.400	78.0	22.0	4
16.00	0.400	65.0	35.0	4
18.00	0.400	45.0	55.0	4
23.00	0.400	0.0	100.0	4
25.00	0.400	98.0	2.0	4
26.00	0.400	98.0	2.0	4

The MS analysis conditions were as follows: The capillary voltage was 3.0 kV Use of high purity nitrogen as atomisation and auxiliary gas and the desolvation gas flow was 800 L/h at a desolvation temperature of 400 ◦C. The sample cone voltage was 40 V, cone gas of 50 L/h, and the source temperature was maintained at 120 ◦C. Sampling frequency was 0.1 s and interval was 0.02 s. Full-scan data were gathered from 50 to 1500 Da in positive mode. The MassLynx software was used to acquire and analyze all data (v4.1, Water, Milford, USA).

2 Structural analysis of compounds in the total extract of *Radix aconiti carmichaeli*

The primary and secondary mass spectra of the constituents of the extract of Aconite were analysed using UPLC/Q-TOF-MS, respectively, and i compounds contained in the samples was determined by reviewing the literature. The ion fragmentation pattern was then analysed according to the ion fragmentation information in the secondary mass spectra and compared with the reference papers。

A total of 23 chemical components including aconitine were identified in the total extract of *Radix aconiti carmichaeli* (**Figure S1)**. The identification results (**Table S2)** and chemical structural formula (**Figure S2)** of specific components are shown in the following figures and tables.

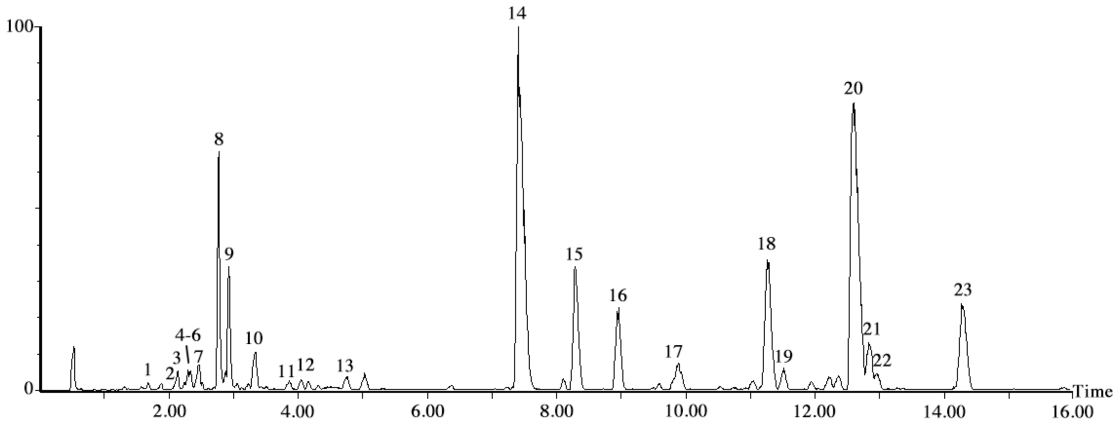

Figure S1 UPLC/Q-TOF-MS analysis (positive ion mode) of the total extract of *Radix aconiti carmichaeli*

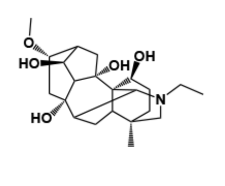

Karakolidine

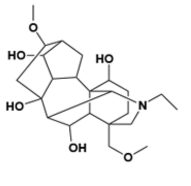

Senbusine A

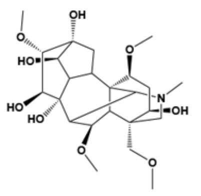

Mesaconine

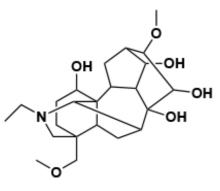

Senbusine B

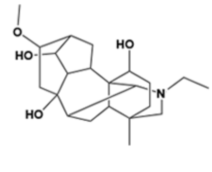

Carmichaeline

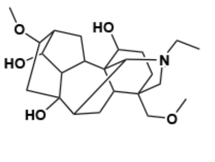

Talatizidine

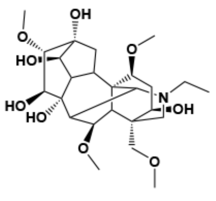

Aconine

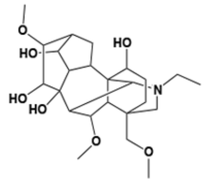

Fuziline

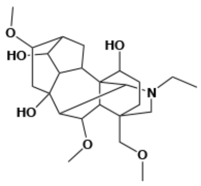

Neoline

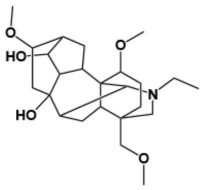

Talatisamine

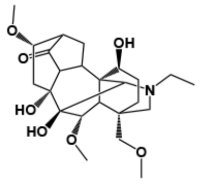

Dehydrodelcosine

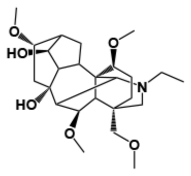

Chasmanine

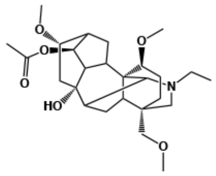

14-O-Acetyltalatizamine

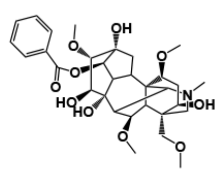

Benzoylmesaconine

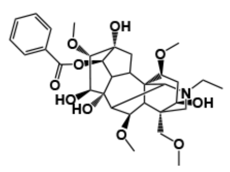

Benzoylaconitine

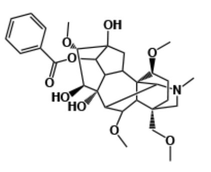

Benzoylhypaconitine

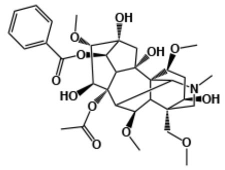

Beiwutine

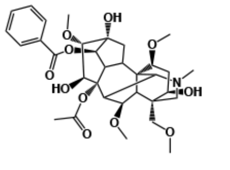

Mesaconitine

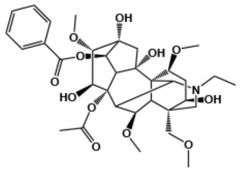

10-hydroxyaconitine

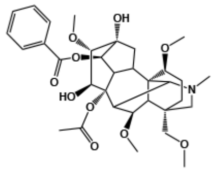

Hypaconitine

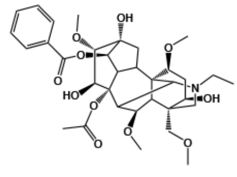

Aconitine

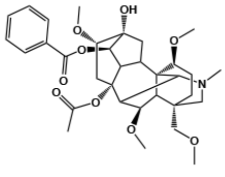

Dexyaconitine

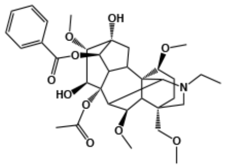

Dexyaconitine

Figure S2 Chemical structure formulae of alkaloid components in the total extract of *Radix aconiti carmichaeli*

Table S2 Analytical table for the identification of alkaloid components in the total extract of *Radix aconiti carmichaeli*

No.	t_R/min	Identification	Exact Mass	*m*/*z*	MS/MS	Composition
1	1.68	Karakolidine	393.52	394.2603	$394[M+H]^+$	$C_{22}H_{35}NO_5$
2	2.06	Senbusine A	423.54	424.2697	$424[M+H]^+$, $374[M+H-CH_3OH-H_2O]^+$,	$C_{23}H_{37}NO_6$
3	2.13	Mesaconine	485.57	486.2687	$486[M+H]^+$, $376[M+H-2CH_3OH-CO-H_2O]^+$	$C_{24}H_{39}NO_9$
4	2.23	Senbusine B	423.54	424.2697	$424[M+H]^+$, $394[M+H-CH_2O]^+$	$C_{23}H_{37}NO_6$
5	2.30	Carmichaeline	377.52	378.2636	$378[M+H]^+$, $360[M+H-H_2O]^+$	$C_{22}H_{35}NO_4$
6	2.33	Talatizidine	407.54	408.2761	$408[M+H]^+$	$C_{22}H_{37}NO_5$
7	2.45	Aconine	499.59	500.2879	$500[M+H]^+$, $408[M+H-2CH_3OH-CO]^+$	$C_{25}H_{41}NO_9$
8	2.77	Fuziline	453.57	454.2811	$454[M+H]^+$, $436[M+H-H_2O]^+$	$C_{24}H_{39}NO_7$
9	2.93	Neoline	437.57	438.2867	$438[M+H]^+$,$420[M+H-H_2O]^+$, $342[M+H-CH_3OH-CO-2H_2O]^+$	$C_{24}H_{39}NO_6$

10	3.34	Talatisamine	421.57	422.2889	422$[M+H]^+$, 344$[M+H-CH_3OH-CO-H_2O]^+$	$C_{24}H_{39}NO_5$
11	3.71	Dehydrodelcosine	451.55	452.3009	452$[M+H]^+$, 438$[M+H-CH_2]^+$	$C_{24}H_{37}NO_7$
12	4.05	Chasmanine	451.59	452.3009	452$[M+H]^+$	$C_{25}H_{41}NO_6$
13	4.75	14-O-Acetyltalatizamine	463.61	464.2999	464$[M+H]^+$	$C_{26}H_{41}NO_6$
14	7.41	Benzoylmesaconine	589.67	590.2942	590$[M+H]^+$, 558$[M+H-CH_3OH]^+$	$C_{31}H_{43}NO_{10}$
15	8.28	Benzoylaconitine	603.70	604.3073	604$[M+H]^+$	$C_{32}H_{45}NO_{10}$
16	8.96	Benzoylhypaconitine	573.67	574.2979	574$[M+H]^+$	$C_{31}H_{43}NO_9$
17	9.9	Beiwutine	647.71	648.3005	648$[M+H]^+$, 588$[M+H-2CH_2O]^+$	$C_{33}H_{45}NO_{12}$
18	11.26	Mesaconitine	631.71	632.3073	632$[M+H]^+$	$C_{33}H_{45}NO_{11}$
19	11.51	10-hydroxyaconitine	661.73	662.3137	662$[M+H]^+$, 586$[M+H-CH_2O-CO-H_2O]$	$C_{34}H_{47}NO_{12}$
20	12.6	Hypaconitine	615.71	616.3057	646$[M+H]^+$	$C_{33}H_{45}NO_{10}$
21	12.85	Aconitine	645.74	646.3190	616$[M+H]^+$	$C_{34}H_{47}NO_{11}$
22	12.95	Delphinine	599.71	600.3139	600$[M+H]^+$, 572$[M+H-CO]^+$	$C_{33}H_{45}NO_9$
23	14.28	Dexyaconitine	629.74	630.3196	630$[M+H]^+$	$C_{34}H_{47}NO_{10}$
